# Epidemiological analysis of structural alterations of the nasal cavity associated with obstructive sleep apnea syndrome (OSA)

**DOI:** 10.1016/S1808-8694(15)31200-3

**Published:** 2015-10-20

**Authors:** Levon Mekhitarian Neto, Antonio Sérgio Fava, Hugo Canhete Lopes, Aldo Stamm

**Affiliations:** 1 Master studies under course.; 2 Faculty, Post-graduation course, Hospital Heliópolis.; 3 Resident Physician in Otorhinolaryngology, Hospital Prof. Edmundo Vasconcelos.; 4 Ph.D. in Otorhinolaryngology, UNIFESP. Study carried out at Hospital Prof. Edmundo Vasconcelos

**Keywords:** apnea, nasal obstruction, septoplasty

## Abstract

**Aim**: The objective of this paper is to demonstrate that structural alterations of the nasal cavity, e.g. septal deviation and conchal hypertrophy have high incidence in patients with sleep apnea and hypopnea syndrome and must be addressed with associated specific procedures of the syndrome. **Type of Study:** Clinical retrospective. **Casuistic and Method**: A retrospective study of 200 patients was performed, with 196 male and 4 female, attended at the otorhinolaryngology ambulatory of Hospital Prof. Edmundo Vasconcelos and Unidade Paulista de Otorrinolaringologia, all of them subjected to polysomnography, otorhinolaryngological physical exam, endoscopy exam, and surgical treatment with nasal and pharyngeal procedures. **Results**: All of them were subjected to pharyngeal procedure: uvulopalatopharyngoplasty or uvulopalatoplasty and nose procedure: 176 septoplasty with partial turbinectomy (88%) and 24 isolated turbinectomy, with satisfactory results. **Conclusion**: We can see that structural alterations of the nasal cavity have high incidence in patients with OSA.

## INTRODUCTION

The obstructive sleep apnea syndrome (OSA) is a chronic and evolution disease presenting high morbidity and mortality rates, affecting 4% of men and 2% of women. It presents a wide range of symptoms, among which we have snoring (90% of cases), increased daytime somnolence, mood changes, daytime headache, sexual impotence, decreased intellectual performance and cardiologic and neurological disorders as the most important signs^1^. Apneas and hypopneas are, respectively, the interruption and reduction of air flow of the upper airways (UA) with minimal duration of 10 seconds, occurring repeatedly and exclusively during sleep.^2^, ^3^ Nasal obstruction is a relatively common problem that may harm quality of life and cause or worsen cases of night apnea. Structural alterations such as septal deviation, traumatic lesions, neoplastic lesions and polyps, fall of nasal valve, adenoid enlargement and presence of foreign bodies are among the main causes of nasal obstruction^4^.

Patient’s evaluation with suspected OSA must include anamnesis comprehending graded scale for snoring and sleepiness. Regarding complementary exams, anterior and posterior rhinoscopy, oroscopy and nasofibrolaryngoscopy with rigid and/or flexible endoscopes to establish the possible site of structural alterations related to the syndrome^5^. Polysomnography, a generic term referring to the simultaneous registry of physiological variables during sleep, such as electroencephalography (EEG), electromyography (EMG), electrocardiogram (ECG), nasal and oral air flow, respiratory effort and blood gases (oxygen saturation and carbon dioxide concentration), is the elective examination to classify OSA. Therefore, added to the number of the respiratory event (called AHR - apnea-hypopnea rate per hour^6^), this is an essential procedure to select the correct approach for each case.

OSA’s treatment may be clinical or surgical and is directly related to AHR, which classifies the disease into mild (AHR 5 to 15), moderate (AHR 15 to 30) and severe (AHR above 30). In patients up to AHR 15, auxiliary methods should be considered important in the treatment of this pathology, as well as behavioral attitudes such as losing weight, avoiding alcohol beverages and sedatives, as well as smoking cessation^7^.

Currently, the most adopted clinical treatment for OSA is mask application, with continuous positive airway pressure (CPAP) bound to an air compressor. Apnea and snoring respond well to CPAP in patients with AHR above 30, once they present immediate improvement and high mask tolerance^7^. Adequacy and comfort of the mask associated with CPAP adequate pressure definition is the most relevant factor for treatment efficacy^8^.

Surgical treatment of nasal structural alterations, such as septoplasty, turbinectomy and polypectomy, offers limited efficiency in the treatment of adults with OSA, although it improves tolerance to CPAP ^9^.

Several surgical approaches have been proposed and the type chosen is related to severity of OSA, as well as to nasal, pharyngeal and craniofacial alterations previously found. The procedures performed include: pharyngeal (tonsillectomies, uvulopalatopharyngoplasty with cautery or laser and palatal radiofrequency); nasal (septoplasty, turbinectomy and conchal cauterization); maxillary-facial (maxillo-mandibular advancement).

The present study aims at demonstrating that structural alterations of the nasal cavity, such as septal deviation and conchal hypertrophy, are highly frequent among OSA patients and should be referred for surgical approaches (septoplasty and/or turbinectomy) associated with other specific procedures of this syndrome.

## METHOD

This is a retrospective study comprehending 200 patients that were assisted at *Hospital Prof. Edmundo Vasconcelos (SP)* and at *Unidade Paulista de Otorrinolaringologia* due to sleep disordered breathing, in the period of March 1992 and December 2003. All patients were submitted to directed anamnesis, comprehensive otorhinolaryngological exam and nasofibroscopy with Muller maneuver and polysomnography. Treatment was indicated after outcome analysis. Other evaluated disorders included cranio-maxillofacial disorders, obesity, body mass index (BMI), arterial hypertension, diabetes mellitus and behavioral habits.

Surgical treatment was indicated based on the structural alterations of the upper airways, facial skeleton and polysomnographic classification, to which apnea-hypopnea rate (AHR) and oxyhemoglobin saturation are greatly relevant. Nasal surgical procedures included septoplasty and/or turbinoplasty, while pharyngeal surgeries included uvulopalatopharyngoplasty, uvulopalatoplasty with cautery or laser.

## RESULTS

Out of 200 patients, 196 were men (98%) and 4 were women (2%) (Table 1) within the age range of 22 and 66 years. Surgeries performed included: uvulopalatopharyngoplasty with septoplasty and turbinoplasty ^5^, uvulopalatoplasty with cautery, with septoplasty and turbinoplasty (161), uvulopalatoplasty with cautery and turbinoplasty^2^, ^4^ and uvulopalatoplasty with laser, plus septoplasty and turbinoplasty^10^.

**Table 1:** 

male	female
196	4

These procedures were grouped according to nasal structural disorders: 176 septoplasties with turbinoplasties (88%) and 24 turbinoplasties (12%) (Table 2).

**Table 2:** 

Septoplasty plus turbinoplasty	turbinoplasty
176	24

## DISCUSSION

Even though some authors^6^ agree that the surgical treatment of nasal structural alterations, such as septal deviation and conchal hypertrophy, offers limited efficiency in treatment of OSA, there are several factors supporting this therapeutic approach. Nasal obstruction and consequent mouth breathing lead to growth and craniofacial development disorders, especially during the first years of life. This fact is due to fast growth of facial structures, particularly of the mid-third of the face, overcoming skull development.

A mouth breather has a lower tongue posture, in close contact with the mouth floor, and low pressure over the palate. This results in high palate and maxillary narrowing and consequent deviation of the upper dental arch, leading to cross bite. Tongue posture disorders, muscular tonus and inadequate dental occlusion also cause suction alterations, atypical chewing and deglutition. Persistence of these disorders in adolescence and adult age is firstly related to important cause of snoring^10^, ^11^ and, secondly, to progressive and persistent apnea and/or hypopnea.

Considering these issues, we believe that those data are relevant in OSA’s genesis, in which nasal obstruction is a very important factor that should be treated.

This study shows evidences of nasal disorders in patients with OSA. All patients (200) were submitted to surgical procedures in order to enhance the retro-palatine space (Table 1) and nasal obstruction was approached simultaneously (176 septoplasties with partial turbinectomies and 24 single turbinectomies), which is a procedure supported by several studies^10^, ^12^.

Another important aspect is that some patients, along OSA’s evolution, even after undergoing surgery, may eventually use CPAP for a more effective control. In addition, surgical treatment may probably improved CPAP tolerance since patients with nasal obstruction have enhanced positive pressure^11^, ^12^, ^13^.

Finally, it is our opinion that diagnostic approach of nasal structural alterations - particularly septal deviation and/or conchal hypertrophy, polyps or other pathology leading to obstruction of natural air flow, is of great relevance in the assessment of patients with suggestive symptomatology of sleep disordered breathing.

Concomitant treatment of these disorders and of OSA has proved to be feasible and, when associated with surgical and/or clinical procedures, such as CPAP, it offers great chances to reach improved outcomes in these patients.
